# Glycolytic reprogramming of resident alveolar macrophages contributes to reduced SOCS3 secretion in non-small cell lung cancer

**DOI:** 10.3389/fimmu.2025.1708467

**Published:** 2026-01-06

**Authors:** Jennifer M. Speth, Mikel D. Haggadone, Marc Peters-Golden

**Affiliations:** 1 Division of Pulmonary and Critical Care Medicine, Department of Internal Medicine, University of Michigan Health System, Ann Arbor, MI, United States; 2 Department of Microbiology, Perelman School of Medicine, University of Pennsylvania, Philadelphia, PA, United States

**Keywords:** alveolar macrophage, extracellular vesicles, glycolysis, lung cancer, SOCS3

## Abstract

Alveolar macrophages (AMs), the resident immune cells of the lung, play a critical role in maintaining pulmonary homeostasis, in part through the secretion of suppressor of cytokine signaling 3 (SOCS3)—a recognized tumor suppressor—within extracellular vesicles (EVs). While we have previously observed that SOCS3 secretion by AMs is diminished in tumor-bearing lungs, the mechanisms underlying this impairment remain unclear. Here, we investigated whether increased glycolytic metabolism in AMs contributes to this defect within the tumor microenvironment. The analysis of published single-cell RNA-sequencing datasets from an orthotopic Lewis lung cancer (LLC) model of adenocarcinoma and non-small cell lung cancer (NSCLC) patients revealed distinct AM clusters in tumor-bearing lungs enriched for glycolysis-associated genes. In a *Kras*^G12D^ mutant mouse model of lung cancer, we found that AMs isolated from tumor-bearing lungs exhibited increased glucose uptake, which inversely correlated with SOCS3 secretion. Importantly, the pharmacologic inhibition of glycolysis with 2-deoxy-d-glucose restored SOCS3 secretion in these AMs. Together, our findings demonstrate that lung tumor-associated AMs undergo a time-dependent metabolic shift toward glycolysis, resulting in impaired SOCS3 secretion—a phenotype that can be reversed by targeting glycolytic flux. These results highlight a potential therapeutic approach for modulating immune suppression in the tumor microenvironment.

## Introduction

Alveolar macrophages (AMs) are essential immune cells residing in the pulmonary alveoli, where they help maintain lung homeostasis and coordinate immune responses to inhaled pathogens and particulates (1). In non-small cell lung cancer (NSCLC), AMs occupy a highly dynamic and complex niche within the tumor microenvironment (2). Emerging research highlights that metabolic reprogramming of AMs is a key mechanism driving immune modulation in the lung (2). Tumor-derived signals such as cytokines (1), metabolites (3, 4), lipids (5), and extracellular vesicles (6) can induce AMs to adopt distinct metabolic states, promoting immunosuppressive phenotypes that support cancer cell transformation (7) and tumor growth (8–11), facilitate angiogenesis (12), and contribute to therapy resistance (11).

Suppressor of cytokine signaling 3 (SOCS3) is a crucial negative regulator of JAK/STAT signaling pathways that mediate cytokine actions and thus plays an important role in modulating inflammation and immune responses (13). Multiple studies have shown that reduced SOCS3 expression is associated with tumor development and progression, including NSCLC (14). Our previous studies support this, demonstrating a loss of secretion of anti-tumorigenic vesicular SOCS3 from AMs isolated from lung tumor-bearing mice (15). However, the underlying mechanism for this defect remains unclear.

In the normal oxygen-rich lung environment, AMs primarily rely on oxidative phosphorylation for the generation of ATP (16) but can switch to glycolysis in the event of perturbations or hypoxia (17, 18). Our more recent studies have shown that in response to growth factors such as granulocyte-macrophage colony-stimulating factor (GM-CSF), AMs increase their glycolytic activity, which leads to a reduction in vesicular SOCS3 secretion. This effect is primarily mediated by ATP citrate lyase (ACLY) and its enzymatic generation of acetyl-CoA (19). Based on these findings, we hypothesized that a pro-glycolytic shift occurs in AMs within tumor-bearing lungs and is responsible for their impaired SOCS3 secretion.

In the present study, we demonstrate that AMs isolated from mice in two models of NSCLC and from NSCLC patients exhibit elevated expression of glycolytic genes and display a glycolytic phenotype, notably through increased expression of genes related to non-canonical tricarboxylic acid (TCA) cycle flux and acetyl-CoA production. In a G12D mutant mouse model of *Kras*-induced adenocarcinoma, we further show that increased glucose uptake by AMs is negatively correlated with SOCS3 secretion, and the inhibition of glycolysis restores their ability to secrete SOCS3.

Most research on the modulation of tumorigenesis by macrophages has targeted tumor-associated macrophages (TAMs). By instead focusing on the metabolic adaptations of resident AMs distributed more broadly throughout a tumor-bearing lung, our work provides novel insight into immune influences governing the susceptibility to tumor development. These findings may ultimately reveal novel therapeutic strategies aimed at improving outcomes for lung cancer patients.

## Methods

### Animals

For experiments with AMs from tumor-bearing lungs, 6–8-week-old LSL-*KRAS*^G12D^ mice or wild-type (WT) littermate controls were used, and experiments were performed as described in our approved protocol (PRO00012746). Animals were treated according to National Institutes of Health (NIH) guidelines for the use of experimental animals, with the approval of the University of Michigan Committee for the Use and Care of Animals, and were maintained at the University of Michigan Unit for Laboratory Animal Medicine. Mutant *Kras* expression was initiated by oropharyngeal (o.p.) administration of Ad5CMV-Cre (2.5 × 10^7^ pfu/mouse). Mice were sacrificed via drop jar inhalation of isoflurane gas at 4 or 16 weeks post-adenoviral infection.

### AM isolation from mouse lungs and *in vitro* culture for detection of SOCS3 in conditioned medium

Primary AMs were collected following lung lavage of mutant or WT *Kras* mice and plated in serum-free Roswell Park Memorial Insititute-1640 (RPMI-1640) at a concentration of 1 × 10^6^ cells/mL, as previously described (15). Briefly, mouse lungs were lavaged several times with 0.5 mL of cold Phosphate buffered saline (PBS), yielding a total of 3 mL of collected fluid. The recovered cells were then centrifuged at 500 × *g* for 5 min at 4 °C and subsequently seeded onto tissue culture-treated plates in serum-free RPMI medium. Following a 1-h incubation to allow for cell adherence, plates were gently washed twice with pre-warmed medium to remove non-adherent cells. This protocol consistently yields AMs with greater than 99% purity, as previously determined via the Wright–Giemsa staining (20). AM conditioned medium (CM) was collected after 24-h culture and subjected to centrifugation at 4°C to remove dead cells/debris (500 × *g* for 5 min) and apoptotic bodies (1,200 × *g* for 10 min). For glycolysis inhibition experiments, AMs were treated with 5 mM 2-deoxy-d-glucose (19, 21) (2-DG; Sigma, St. Louis, MO, USA) for 24 h before collection of CM.

### SOCS3 ELISA

Mouse AM CM was harvested as described above, and SOCS3 levels were determined using a mouse SOCS3 ELISA kit (Cloud-Clone Corp, Katy, TX, USA) after brief sonication to disrupt extracellular vesicles (EVs), as described previously (15).

### Glucose uptake assay

Glucose uptake in AMs was measured using the Glucose Uptake-Glo assay (Promega, Madison, WI, USA). Briefly, AMs were isolated by lung lavage from WT and *Kras* mutant mice at 4 and 16 weeks following adenoviral infection, as described above. The cells were seeded into 96-well tissue culture plates at a density of approximately 5,000 cells per well and allowed to adhere for 1 h. Subsequently, non-adherent cells were removed by washing twice with pre-warmed medium. Glucose uptake was then assessed according to the manufacturer’s instructions.

### RNA isolation and glycolysis PCR array

The analysis of gene transcript levels was performed by extracting total RNA using an RNeasy kit (Qiagen, Germantown, MD, USA). cDNA was prepared using a High-Capacity cDNA Reverse-transcription kit (Applied Biosystems, Foster City, CA, USA). Glycolysis gene mRNA levels were determined using the Mouse Glucose Metabolism RT (2) Profiler™ PCR Array (Qiagen) and a StepOne thermocycler (Applied Biosystems), or by SYBR Green quantitative real-time PCR. Relative gene expression was determined using the ΔCT method, with GAPDH as a reference gene (gene list and primer sequences listed in **Supplementary Table S1**).

### Reanalysis of published mouse and human AM single-cell RNA-sequencing data

For Lewis lung cancer (LLC) model analysis, single-cell RNA-sequencing (scRNA-seq) datasets were obtained from AMs isolated from LLC tumor-bearing mice (GSE193914) (9) from the Gene Expression Omnibus. Data were analyzed using the Seurat v5 workflow in R Studio (R version 4.4.1). For quality control, genes detected in fewer than three cells in a sample, cells with <200 or >7,500 genes, and cells with >10% mitochondrial genes were removed. Data were then integrated using scTransform and clustered using the standard Seurat Louvain method (22). Pathway and gene set enrichment analyses were performed using the SeuratExtend (v1.2.1) package using the GO Hallmark and GO Glycolytic Process database (23). Additional testing for glycolysis gene enrichment was performed using the AddModuleScore feature within Seurat and a curated gene set based on known glycolytic genes (**Supplementary Table S2**). For human NSCLC patient analysis (GSE136246) (24), 12 separate Red blood cells (RBC)-depleted tumor digests (one per patient) were compared to a normal lung dataset (Human Lung Cell Atlas, V1) (25). The 13 datasets were merged into a combined Seurat object for comparative analysis. Pre-integration clustering was conducted using the first 15 principal components at a resolution of 0.3. Integration was then performed using Harmony with FindIntegrationAnchors() on SCT-normalized data using the top 3,000 variable features. Integrated embeddings from Harmony were used for downstream marker discovery and cell type annotation. Cell type annotation was conducted manually using known cell markers. Macrophage subsets were reanalyzed using the same Seurat workflow as above.

### Statistical analysis

Data are expressed as mean ± SEM from at least three independent experiments unless noted otherwise in the figure legend. Data were analyzed using the Prism 5.0 statistical program from the GraphPad software, using either ANOVA with Bonferroni *post-hoc* correction or Student’s t-test, as appropriate. For correlation analysis, Spearman’s rank correlation was used. Statistical significance was set at p-value <0.05.

## Results

### AMs isolated from LLC tumor-bearing lungs include enriched subsets exhibiting enhanced glycolytic gene signatures compared to AMs isolated from normal lungs

**Figure 1:**
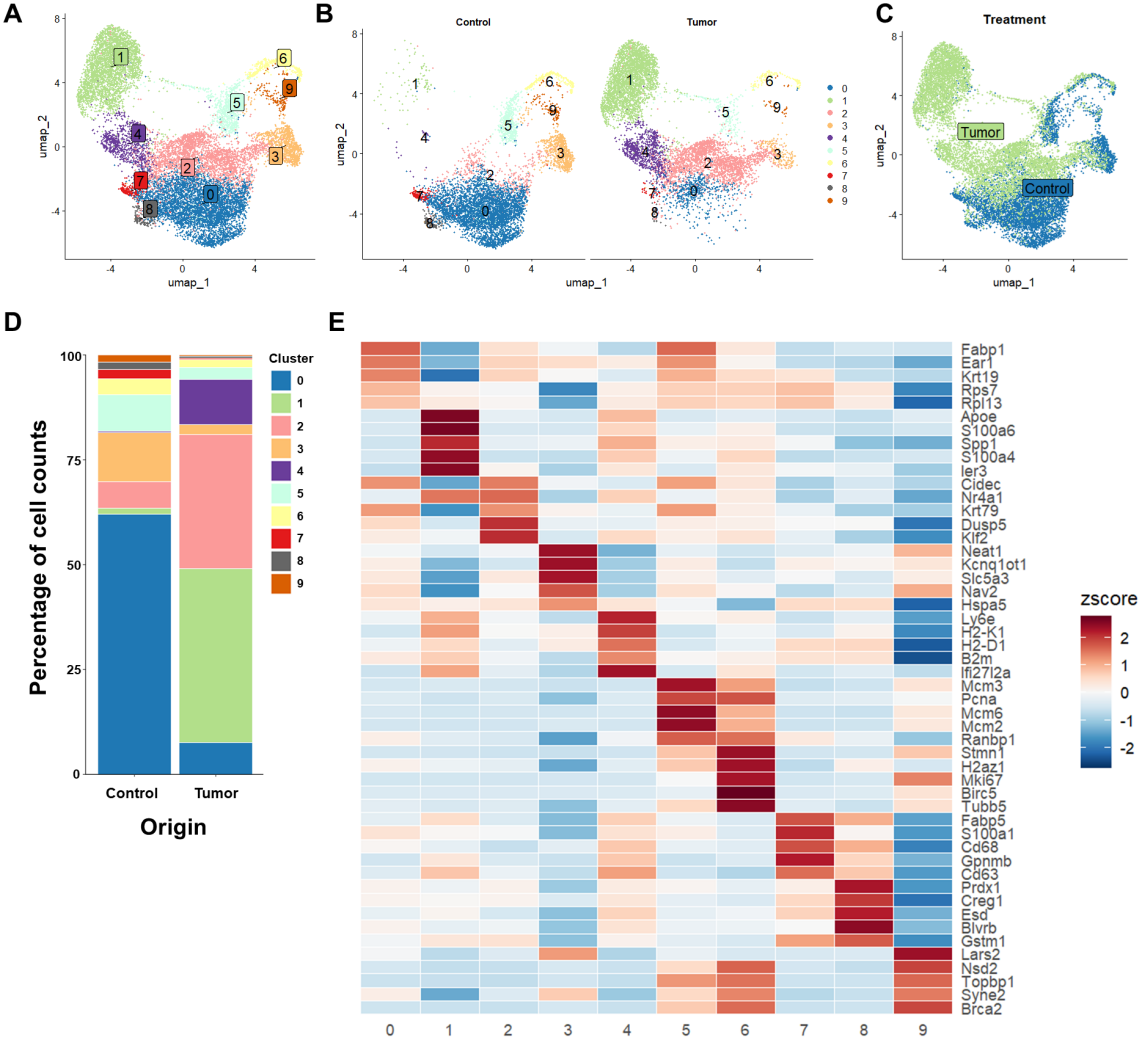
Single-cell RNA-sequencing analysis of alveolar macrophages (AMs) isolated from Lewis lung cancer (LLC) tumor-bearing lungs. **(A)** Fully integrated uniform manifold approximation and projection (UMAP) of sorted AMs isolated from tumor-bearing lungs and normal lungs. **(B)** UMAP of AM clusters from tumor-bearing lungs *vs*. control lungs. **(C)** UMAP overlay of AMs isolated from tumor-bearing lungs *vs*. control lungs. **(D)** Proportion of total AMs within specific clusters for tumor-bearing and control lungs. **(E)** Top 5 differentially expressed genes for each cluster (based on significance threshold of p < 0.05).

**Figure 2:**
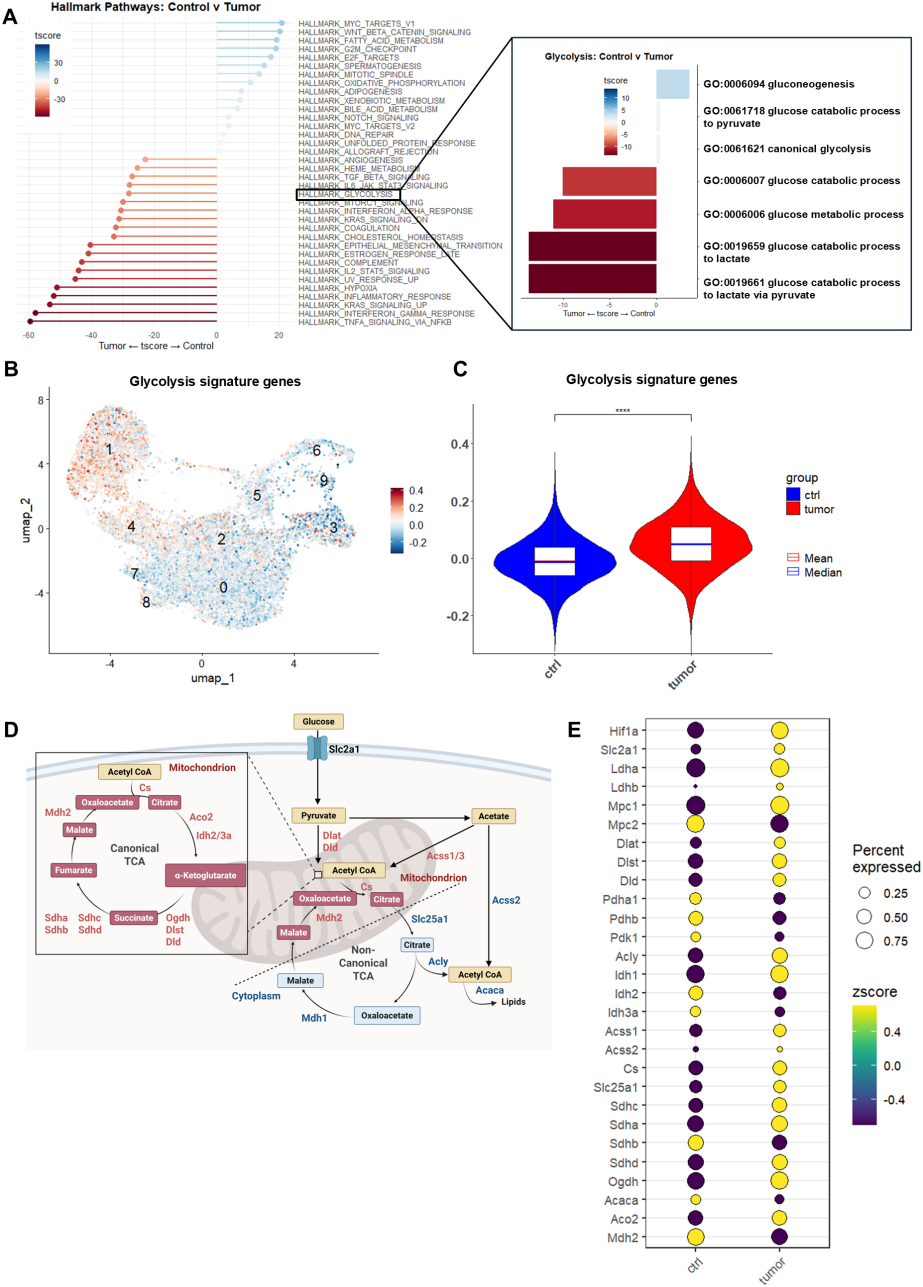
Alveolar macrophages (AMs) from Lewis lung cancer (LLC) tumor-bearing lungs are enriched for genes associated with glycolysis and alternative tricarboxylic acid (TCA) cycle. **(A)** Waterfall plot showing hallmark pathways enriched in tumor AMs *vs*. control AMs. (Inset) Gene set enrichment analysis (GSEA) waterfall plot of Gene Ontology (GO) pathways specific to glycolysis comparing tumor AMs to control AMs. **(B)** FeaturePlot displaying the distribution of module scores for glycolysis signature genes (**Supplementary Table S2**). Cells exhibiting higher scores (i.e., those expressing more glycolysis signature genes) are shown in red. **(C)** Violin plot of glycolysis signature gene module scores comparing tumor AMs to control AMs. **(D)** Diagram describing genes associated with canonical (red) *vs*. non-canonical (blue) TCA cycle. **(E)** Dot plot showing relative expression of genes associated with the TCA cycle for both tumor and control AMs, where color represents relative expression levels and circle size represents proportion of cells expressing each gene. ****p < 0.0001.

To determine whether AMs residing in tumor-bearing lungs exhibited gene expression patterns consistent with enhanced glycolysis, we reanalyzed a previously published scRNA-seq dataset in which Siglec-F^+^ resident AMs were sorted from the lungs of mice orthotopically inoculated with LLC cells (9). Using uniform manifold approximation and projection (UMAP) analysis, we subclustered the cells into 10 groups (**Figure 1A**). Consistent with previous findings (9), we found three distinct clusters of AMs present only or predominantly in the tumor-bearing lungs compared to normal lungs (**Figures 1B–D**). Cluster 1 expressed *S100a4*, *S100a6*, and *Spp1*, genes associated with tumor progression and metastasis (12, 26–28). Cluster 2 expressed *Nr4a1*, a gene associated with unique glycolytic regulating effects in tumor cells (29). Cluster 4 expressed elevated levels of *B2m* and MHC-associated genes, suggesting that the activation of a subset of AMs may have anti-tumor capacity (30, 31) (**Figure 1E**). Gene set enrichment analysis revealed enrichment in glycolysis-associated genes in the tumor AMs compared to control (**Figure 2A**), which was primarily localized in the unique clusters only present in the tumor-bearing lung (**Figures 2B, C**). Interestingly, the proportion of cells within cluster 0, which expressed genes characteristic of “traditional” resident AMs, such as *Fabp1* and *Ear1*, was diminished in the tumor-associated AMs. This reduction may indicate a shift from a homeostatic AM population to the tumor-specific populations identified in clusters 1 and 2. Alternatively, it could reflect an increase in apoptosis among resident AMs, leading to their replacement by recruited macrophages that are phenotypically shaped by the tumor microenvironment.

### AMs from LLC lungs are enriched for genes associated with acetyl-CoA production via alternative TCA flux

Typically, after glucose enters the cell and is metabolized to pyruvate, it is transported into the mitochondria and converted to acetyl-CoA. This acetyl-CoA then enters the canonical TCA cycle, where it is oxidized to produce ATP and generate precursors for various biosynthetic pathways. Alternatively, citrate produced in the mitochondria can be exported back to the cytoplasm and converted to acetyl-CoA, or acetyl-CoA can be generated through the conversion of acetate (32) (**Figure 2D**). These alternative metabolic routes, which occur via non-canonical TCA cycle pathways, are often associated with disease states such as cancer (33). Our previous studies demonstrated that under glycolytic conditions, the ability of AMs to package SOCS3 into extracellular vesicles was impaired, likely due to the increased conversion of citrate into acetyl-CoA through non-canonical TCA flux (19). To further investigate this, we compared the expression levels of genes involved in the non-canonical TCA cycle in AMs isolated from LLC-bearing lungs versus control lungs. Notably, we found that AMs from tumor-bearing lungs exhibited increased expression of genes related to citrate synthesis and transport (*Cs* and *Slc25a1*, respectively), cytoplasmic conversion of citrate to acetyl-CoA (*Acly*), and acetate conversion to acetyl-CoA (*Acss1/2*) in addition to traditional glycolytic genes such as glucose transporters (*Slc2a1*), mitochondrial pyruvate carriers (*Mpc1*), and hypoxia-inducible factors (*Hif1a*) (**Figure 2E**). Taken together, these data suggest that in an orthotopic mouse model of LLC, AMs that are present in tumor-bearing lungs exhibit an enhanced glycolytic phenotype and appear to have increased non-canonical TCA flux. This, we reasoned, could result in decreased vesicular SOCS3 packaging/secretion and thereby contribute to tumor development and progression.

### Reduced AM SOCS3 secretion correlates with a glycolytic phenotype in a *Kras* mutant lung cancer model

**Figure 3:**
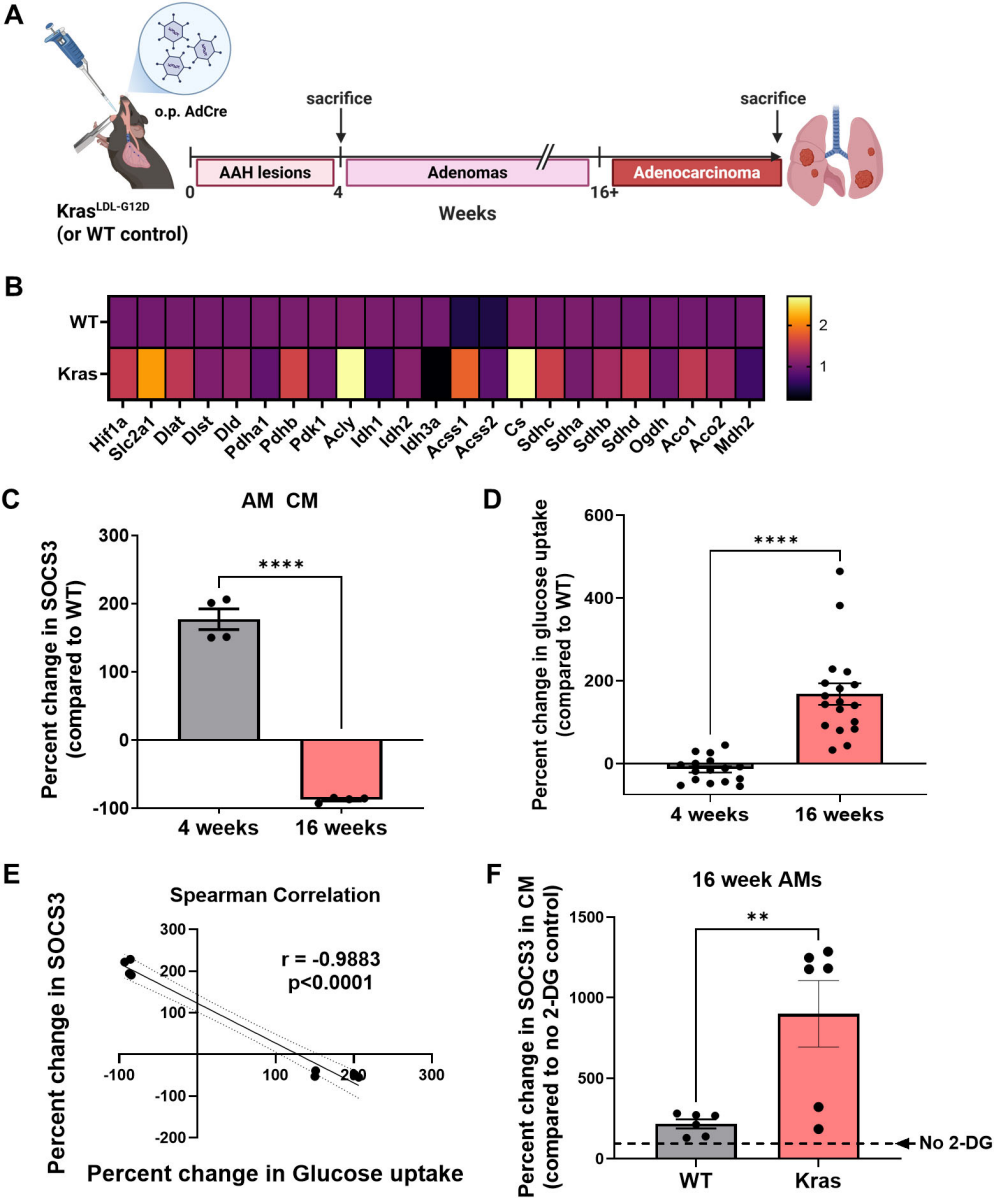
In a mutant Kras model, alveolar macrophage (AM) suppressor of cytokine signaling 3 (SOCS3) secretion is negatively correlated with glucose uptake and is enhanced by inhibition of glycolysis. **(A)** Experimental timeline illustrating induction of mutant *Kras* tumors via adenoviral Cre recombinase administration and mouse harvest. **(B)** Heatmap of relative expression levels of glycolysis and tricarboxylic acid (TCA)-associated genes for AMs isolated from *Kras* mutant and wild-type (WT) lungs. **(C)** Quantification of SOCS3 levels in AM conditioned medium (CM) from *Kras* mutant mice, expressed as percent change relative to WT controls at 4 and 16 weeks following Cre induction. Each point represents an individual mouse. **(D)** Quantification of glucose uptake in AMs isolated from *Kras* mutant lungs, expressed as percent change compared to WT controls at 4 and 16 weeks following Cre induction. Each point represents an individual mouse. **(E)** Analysis of paired samples (n = 4 mice) assessing the correlation between the percent change in SOCS3 and the percent change in glucose uptake by AMs in 4-week (blue squares) and 16-week (red circles) *Kras* mutant mice. **(F)** Quantification of SOCS3 levels in AM CM from *Kras* mutant mice and WT controls after 2-deoxy-d-glucose (2-DG) treatment (5 mM), expressed as percent change compared to non-treated controls (dashed line) (n = 6 mice). For correlation experiments, n = 4 mice were used for both SOCS3 secretion and glucose uptake. For glucose uptake experiments, an additional n = 11–13 mice were used. ****p < 0.0001; **p < 0.01.

The analysis of the published LLC model dataset strongly pointed to glycolytic reprogramming in AMs from tumor-bearing lungs, but did not allow for the evaluation of their ability to secrete SOCS3 protein within EVs. Therefore, to directly assess whether enhanced glycolysis in AMs disrupts SOCS3 secretion, we utilized a similar but distinct mouse model of NSCLC involving the induction of G12D mutant *Kras* as previously described (15) (**Figure 3A**). We isolated AMs from *Kras* mutant lungs at 4 and 16 weeks post-adenovirus induction and assessed their glycolytic gene expression via PCR array. We selected these specific timepoints based on our prior studies to enable comparison between AMs from lungs bearing established tumors (16 weeks) and those from an earlier stage of tumorigenesis prior to the formation of adenomas and solid tumors (4 weeks) (15). Array analysis revealed a similar pattern of gene expression in AMs from *Kras* mutant lungs harboring tumors as found in those isolated from LLC mice, in which genes associated with glycolysis and non-canonical TCA cycle activity were elevated (**Figure 3B**). We next assessed the ability of these cells to secrete SOCS3 *in vitro*. AMs derived from tumor-bearing lungs exhibited significantly reduced SOCS3 secretion compared to those from non-tumor-bearing lungs (**Figure 3C**). We then measured cellular glucose uptake and found that AMs from tumor-bearing lungs displayed increased glucose uptake relative to their non-tumor counterparts (**Figure 3D**). Correlation analysis revealed a strong inverse relationship between SOCS3 secretion and glucose uptake in AMs (**Figure 3E**). To determine whether elevated glycolysis is mechanistically responsible for the impaired SOCS3 secretion by AMs from tumor-bearing lungs, we treated the cells with the unmetabolizable glucose analog 2-DG to inhibit glycolysis and subsequently measured SOCS3 levels in their CM. Indeed, deficient SOCS3 secretion was rescued in AMs treated with 2-DG (**Figure 3F**). Collectively, these data indicate that the loss of SOCS3 secretion by AMs is dependent on their adoption of a glycolytic phenotype and can be reversed by inhibiting glycolysis. Moreover, this metabolic phenotype is conserved across two distinct models of NSCLC.

### Metabolic reprogramming in AMs is conserved between NSCLC patients and murine models

**Figure 4:**
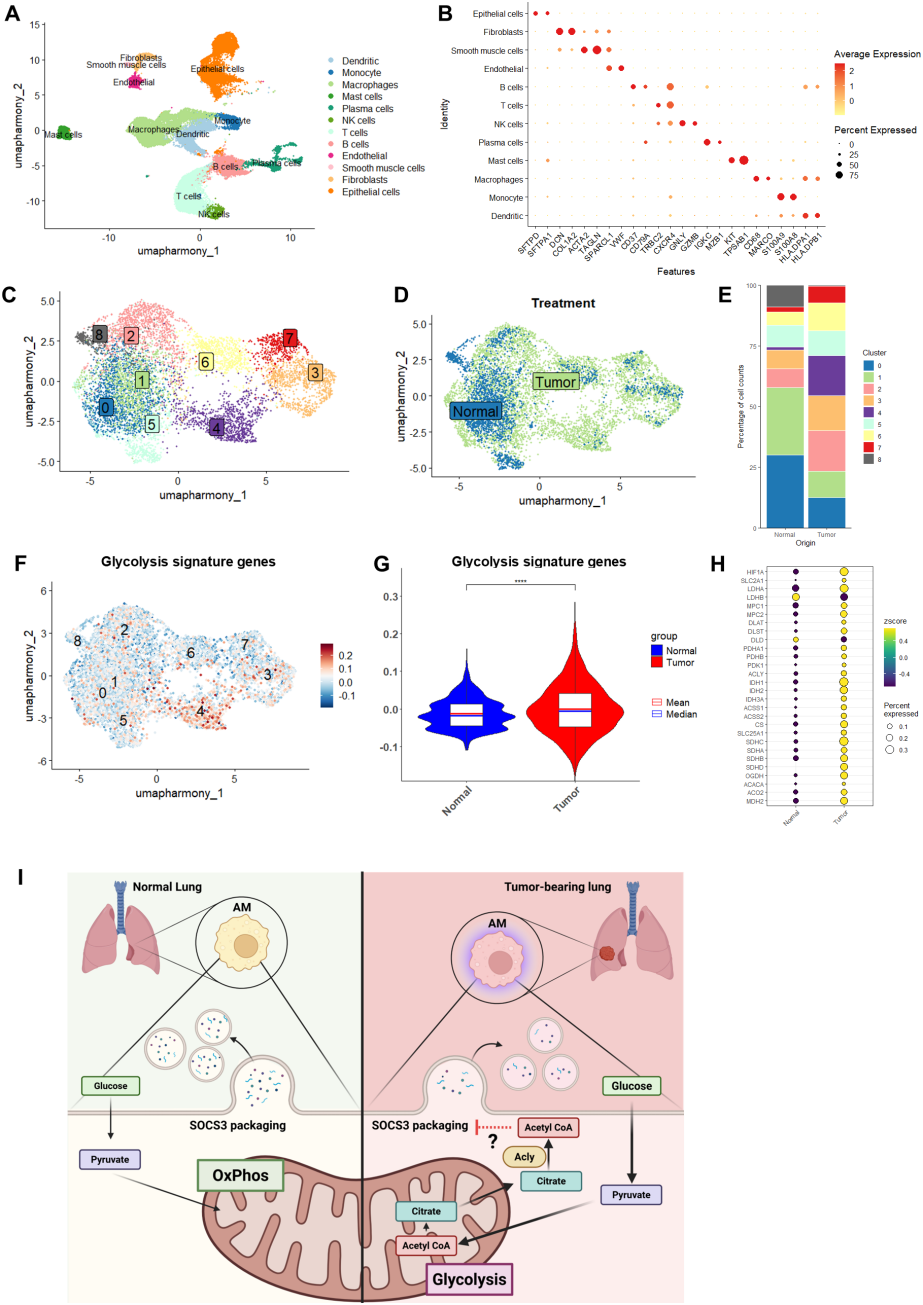
Alveolar macrophages (AMs) from non-small cell lung cancer (NSCLC) patients have enhanced glycolytic and non-canonical tricarboxylic acid (TCA) gene expression. **(A)** Uniform manifold approximation and projection (UMAP) of coarse cell types from normal lung and 12 patient NSCLC tumor digests. **(B)** Dot plot of cell marker gene expression for each cell type. **(C)** Unsupervised clustering of macrophages revealed eight unique clusters. **(D)** Fully integrated UMAP of macrophages from normal and NSCLC (tumor) tissues. **(E)** Cluster bar of cell proportions within macrophage clusters from normal and tumor samples. **(F)** FeaturePlot displaying the distribution of module scores for glycolysis signature genes (**Supplementary Table S2**). Cells exhibiting higher scores (i.e., those expressing more glycolysis signature genes) are shown in red. **(G)** Violin plot of glycolysis signature gene module scores comparing macrophages from NSCLC tumors to normal lung. **(H)** Dot plot showing relative expression of genes associated with the TCA cycle for both tumor and normal macrophages, where color represents relative expression levels and circle size represents proportion of cells expressing each gene. **(I)** Schematic showing enhanced glycolytic and non-canonical TCA cycle activity in AMs from tumor-bearing lungs leads to decreased suppressor of cytokine signaling 3 (SOCS3) packaging into extracellular vesicles (EVs) and reduced secretion due to elevated intracellular acetyl-CoA accumulation. ****p < 0.0001.

To determine whether the upregulation of glycolytic and non-canonical TCA cycle gene expression observed in the *Kras* mutant mouse model is conserved in human cancer, we performed a detailed analysis of a scRNA-seq dataset comparing AMs from 12 NSCLC patient tumor samples to those from normal lung tissue (24, 25). We manually annotated major cell types using established cell markers, revealing a diverse population that included dendritic cells, monocytes, macrophages, mast cells, plasma cells, NK cells, T cells, B cells, endothelial cells, smooth muscle cells, fibroblasts, and epithelial cells (**Figures 4A, B**). Subsequent subclustering of CD68^+^CD206^+^MARCO^+^ AMs identified eight distinct clusters present across both normal and tumor tissue (**Figures 4C, D**). With the exception of clusters 0, 1, and 8, the remaining clusters were more abundant in tumor samples than in normal lung (**Figure 4E**). Notably, cluster 4 displayed a pronounced glycolytic gene signature (**Figures 4F, G**) as well as elevated expression of non-canonical TCA cycle genes (**Figure 4H**), strongly resembling the metabolic phenotype observed in AMs from our murine lung cancer models.

## Discussion

This study provides novel insights into the phenotypic alterations seen in resident AMs within the tumor microenvironment of lung cancer. We found that LLC tumor-bearing lungs exhibited distinct shifts in AM subpopulations. Notably, unique AM clusters emerged in tumor-bearing lungs—some expressing factors associated with tumor progression and others with potential anti-tumor activities—highlighting the heterogeneity and functional plasticity of AMs in this context. Furthermore, the subpopulations appearing *de novo* in tumor-bearing lungs were enriched in glycolytic gene signatures, including the upregulation of key genes involved in glucose uptake and metabolism.

In addition to enhanced glycolytic activity, tumor-associated AMs displayed increased expression of genes involved in alternative, non-canonical TCA cycle pathways, such as those mediating citrate export out of the mitochondria and conversion of citrate and acetate to acetyl-CoA. Importantly, our findings implicate these metabolic changes in the disruption of SOCS3 secretion, a process critical for immune regulation and tumor surveillance (**Figure 4I**). Using a complementary *Kras*-driven model of lung cancer, we found that the glycolytic shift in AMs correlates with a marked reduction in SOCS3 secretion. The strong inverse relationship between glucose uptake and SOCS3 output in AMs, together with the restoration of SOCS3 secretion upon glycolysis inhibition with 2-DG, provides compelling evidence that enhanced glycolysis directly impairs the immunoregulatory function of AMs. This also supports our previous studies that linked the inhibition of SOCS3 packaging to shifts in glycolytic flux and the generation of acetyl-CoA in response to GM-CSF, a growth factor that is itself known to be elevated in tumor-bearing lungs and can promote immune cell evasion by tumor cells (34). These observations are also particularly noteworthy because, unlike recent studies that emphasize how EVs can modulate tumor development and progression by altering cellular metabolism (35), our findings reveal the converse: metabolic changes within cells play a direct role in shaping the protein composition of EV cargo. This distinction highlights a previously underappreciated, bidirectional relationship between cellular metabolism and EV-mediated communication.

Moreover, while our current data support a causal relationship between enhanced glycolytic flux, increased activity of ACLY, generation of acetyl-CoA, and the regulation of vesicular SOCS3 secretion, a critical next step in this field will be to comprehensively determine the exact mechanism by which EV packaging is influenced by acetyl-CoA. The significance of acetyl-CoA extends well beyond energy metabolism; as a key substrate for protein acetylation, it has the potential to modulate diverse cellular processes that impact EV cargo selection. Recent proteomic analyses of exosomes derived from epithelial ovarian cancer cell lines have revealed a notable enrichment of acetylated proteins within these vesicles (36). This observation suggests that acetyl-CoA may positively regulate the packaging of certain proteins into exosomes, potentially through acetylation-dependent mechanisms. However, this relationship is not uniformly positive. For example, acetylation has been shown to inhibit the sorting of glucose-regulated protein 78 (GRP78) into exosomes by promoting its retention in the endoplasmic reticulum (37). These findings indicate that the influence of acetyl-CoA on EV composition may be both cargo-specific and context-dependent, with acetylation capable of either promoting or restricting inclusion of particular molecules within vesicle populations. The complexity of this regulatory network highlights the need for further investigation into the mechanisms by which acetyl-CoA modulates EV cargo processing. Factors such as the local metabolic state, cell type, and the repertoire of available acetylation targets may collectively influence whether acetyl-CoA exerts a positive or negative effect on EV cargo selection.

These findings also illuminate the critical role of metabolic programming in dictating the anti-tumor or pro-tumor functions of innate immune cells (38). By linking glycolytic reprogramming to impaired SOCS3 secretion, our study suggests that targeting metabolic pathways in AMs may represent a novel therapeutic approach to reinvigorate their tumor-suppressive functions. The pharmacologic inhibition of glycolysis, for instance, may restore SOCS3-mediated immunoregulation and limit tumor progression. Furthermore, the identification of non-canonical TCA cycle activity opens additional avenues for metabolic intervention, potentially allowing for the selective targeting of tumor and tumor-associated immune cells while sparing normal tissue function. Indeed, current studies are exploring the *in vivo* therapeutic potential of targeting glycolytic pathways (39–41), and future studies should investigate whether these treatments can also reprogram the immune system to adopt an anti-tumor phenotype. Alternatively, the *ex vivo* direct modulation of macrophages has emerged as an exciting therapeutic option (42). By metabolically reprogramming macrophages *ex vivo* and reintroducing them into the tumor microenvironment, it may be possible to restore their anti-tumor functions and enhance immune-mediated tumor control.

The conservation of the glycolytic phenotype of AMs across both LLC and *Kras* mutant models, as well as among NSCLC patients with *KRAS* mutations, significantly strengthens the validity of our findings and enhances their relevance to human disease. While murine models provide valuable mechanistic insights into the regulation of SOCS3 secretion by metabolic reprogramming, it is essential to establish that these processes are conserved in the human context. Our analyses reveal that AMs from both mouse models of lung cancer and human NSCLC patients exhibit similar enrichment of glycolytic and non-canonical TCA cycle gene signatures, alongside reduced SOCS3 secretion (15). This cross-species agreement suggests that the metabolic mechanisms shaping SOCS3 secretion are evolutionarily conserved and likely operate in clinical disease settings. Such a parallel not only supports the translational significance of our study but also underscores the potential for targeting metabolic pathways to modulate SOCS3 secretion therapeutically in human lung cancer. Ultimately, this integrative approach bridges fundamental research and clinical application, providing a more robust foundation for future studies and potential interventions. Nonetheless, to fully delineate the clinical relevance and generalizability of these findings, it will be important to extend these analyses to include cells harboring additional oncogenic drivers of tumorigenesis, such as EGFR mutations. Examining models characterized by various genetic alterations will further elucidate the mechanistic role of glycolysis in SOCS3 secretion, its impact on lung tumor development, and the broader relevance of our findings.

In summary, our work highlights how the metabolic state of AMs in lung cancer is intimately linked to their functional output and immunoregulatory capacity. Targeting these metabolic adaptations represents a promising strategy to modulate the tumor microenvironment and improve outcomes for patients with NSCLC.

## Data Availability

The raw data supporting the conclusions of this article will be made available by the authors, without undue reservation.
