# Supplementary material for: Glycolytic reprogramming of resident alveolar macrophages contributes to reduced SOCS3 secretion in non-small cell lung cancer
Source: Front Immunol. 2026 Jan 6;16:1708467. doi: 10.3389/fimmu.2025.1708467 (PMC12816181; doi:10.3389/fimmu.2025.1708467)
Supplement: Supplementary file 1 [file DataSheet1.pdf]

Speth, et. al Supplementary Table 1

<table>
<tr><th>Gene Symbol</th><th>Gene Name</th><th>Forward Primer</th><th>Reverse Primer</th></tr>
<tr><td>Hif1α</td><td>Hypoxia inducible factor 1 alpha</td><td>GTCCCAGCTACGAAGTTACAGC</td><td>CAGTGCAGGATACACAAGGTTT</td></tr>
<tr><td>Slc2a1</td><td>Solute carrier family 2, facilitated glucose transporter member 1</td><td>GAGGCATCGAAATCTGCATCA</td><td>GGATGGAGCCGTAGAGCAA</td></tr>
<tr><td>Acss1</td><td>Acetyl-coA synthetase 1</td><td>GTTTGGGACACTCCTTACCATAC</td><td>AGGCAGTTGACAGACACATTC</td></tr>
<tr><td>Acss2</td><td>Acetyl-coA synthetase 2</td><td>AAACACGCTCAGGGAAAATCA</td><td>ACCGTAGATGTATCCCCCAGG</td></tr>
<tr><td>Aldoa</td><td>Aldolase A, fructose-bisphosphate</td><td rowspan="24" colspan="2">RT^2 Mouse Glycolysis PCR Array</td></tr>
<tr><td>Aldob</td><td>Aldolase B, fructose-bisphosphate</td></tr>
<tr><td>Aldoc</td><td>Aldolase C, fructose-bisphosphate</td></tr>
<tr><td>Bpgm</td><td>2,3-bisphosphoglycerate mutase</td></tr>
<tr><td>Eno1</td><td>Enolase 1, alpha non-neuron</td></tr>
<tr><td>Eno2</td><td>Enolase 2, gamma neuronal</td></tr>
<tr><td>Eno3</td><td>Enolase 3, beta muscle</td></tr>
<tr><td>Galm</td><td>Galactose mutarotase</td></tr>
<tr><td>Gapdhs</td><td>Glyceraldehyde-3-phosphate dehydrogenase, spermatogenic</td></tr>
<tr><td>Gck</td><td>Glucokinase</td></tr>
<tr><td>Gpi1</td><td>Glucose phosphate isomerase 1</td></tr>
<tr><td>Hk2</td><td>Hexokinase 2</td></tr>
<tr><td>Hk3</td><td>Hexokinase 3</td></tr>
<tr><td>Pfkl</td><td>Phosphofructokinase, liver, B-type</td></tr>
<tr><td>Pgam2</td><td>Phosphoglycerate mutase 2</td></tr>
<tr><td>Pgk1</td><td>Phosphoglycerate kinase 1</td></tr>
<tr><td>Pgk2</td><td>Phosphoglycerate kinase 2</td></tr>
<tr><td>Pgm1</td><td>Phosphoglucomutase 1</td></tr>
<tr><td>Pgm2</td><td>Phosphoglucomutase 2</td></tr>
<tr><td>Pgm3</td><td>Phosphoglucomutase 3</td></tr>
<tr><td>Pklr</td><td>Pyruvate kinase liver and red blood cell</td></tr>
<tr><td>Tpi1</td><td>Triosephosphate isomerase 1</td></tr>
<tr><td>Fbp1</td><td>Fructose bisphosphatase 1</td></tr>
<tr><td>Fbp2</td><td>Fructose bisphosphatase 2</td></tr>
<tr><td>G6pc</td><td>Glucose-6-phosphatase, catalytic</td></tr>
</table>

G6pc3	Glucose 6 phosphatase, catalytic, 3	
Pck1	Phosphoenolpyruvate carboxykinase 1, cytosolic	
Pck2	Phosphoenolpyruvate carboxykinase 2 (mitochondrial)	
Pcx	Pyruvate carboxylase	
Pdp2	Pyruvate dehyrogenase phosphatase catalytic subunit 2	
Pdpr	Pyruvate dehydrogenase phosphatase regulatory subunit	
Pdk1	Pyruvate dehydrogenase kinase, isoenzyme 1	
Pdk2	Pyruvate dehydrogenase kinase, isoenzyme 2	
Pdk3	Pyruvate dehydrogenase kinase, isoenzyme 3	
Pdk4	Pyruvate dehydrogenase kinase, isoenzyme 4	
Acly	ATP citrate lyase	
Aco1	Aconitase 1	
Aco2	Aconitase 2, mitochondrial	
Cs	Citrate synthase	
Dlat	Dihydrolipoamide S-acetyltransferase (E2 component of pyruvate dehydrogenase complex)	
Dld	Dihydrolipoamide dehydrogenase	
Dlst	Dihydrolipoamide S-succinyltransferase (E2 component of 2-oxo-glutarate complex	
Fh1	Fumarate hydratase 1	
Idh1	Isocitrate dehydrogenase 1 (NADP+), soluble	
Idh2	Isocitrate dehydrogenase 2 (NADP+), mitochondrial	
Idh3a	Isocitrate dehydrogenase 3 (NAD+) alpha	
Idh3b	Isocitrate dehydrogenase 3 (NAD+) beta	
Idh3g	Isocitrate dehydrogenase 3 (NAD+), gamma	
Mdh1	Malate dehydrogenase 1, NAD (soluble	

Mdh1b	Malate dehydrogenase 1B, NAD (soluble)	
Mdh2	Malate dehydrogenase 2, NAD (mitochondrial)	
Ogdh	Oxoglutarate dehydrogenase (lipoamide)	
Pdha1	Pyruvate dehydrogenase E1 alpha 1	
Pdhb	Pyruvate dehydrogenase (lipoamide) beta	
Sdha	Succinate dehydrogenase complex, subunit A, flavoprotein (Fp)	
Sdhb	Succinate dehydrogenase complex, subunit B, iron sulfur (Ip)	
Sdhc	Succinate dehydrogenase complex, subunit C, integral membrane protein	
Sdhd	Succinate dehydrogenase complex, subunit D, integral membrane protein	
Sucla2	Succinate-Coenzyme A ligase, ADP-forming, beta subunit	
Suclg1	Succinate-CoA ligase, GDP-forming, alpha subunit	
Suclg2	Succinate-Coenzyme A ligase, GDP-forming, beta subunit	
G6pdx	Glucose-6-phosphate dehydrogenase X-linked	
H6pd	Hexose-6-phosphate dehydrogenase (glucose 1-dehydrogenase)	
Prps1	Phosphoribosyl pyrophosphate synthetase 1	
Prps1l1	Phosphoribosyl pyrophosphate synthetase 1-like 1	
Prps2	Phosphoribosyl pyrophosphate synthetase 2	
Rbks	Ribokinase	
Rpe	Ribulose-5-phosphate-3-epimerase	
Rpia	Ribose 5-phosphate isomerase A	
Taldo1	Transaldolase 1	
Tkt	Transketolase	

Gbe1	Glucan (1,4-alpha-), branching enzyme 1	
Gys1	Glycogen synthase 1, muscle	
Gys2	Glycogen synthase 2	
Ugp2	UDP-glucose pyrophosphorylase 2	
Agl	Amylo-1,6-glucosidase, 4-alpha-glucanotransferase	
Pygl	Liver glycogen phosphorylase	
Pygm	Muscle glycogen phosphorylase	
Gsk3a	Glycogen synthase kinase 3 alpha	
Gsk3b	Glycogen synthase kinase 3 beta	
Phka1	Phosphorylase kinase alpha 1	
Phkb	Phosphorylase kinase beta	
Phkg1	Phosphorylase kinase gamma 1	
Phkg2	Phosphorylase kinase, gamma 2 (testis)	
