# Supplementary material for: Glycolytic reprogramming of resident alveolar macrophages contributes to reduced SOCS3 secretion in non-small cell lung cancer
Source: Front Immunol. 2026 Jan 6;16:1708467. doi: 10.3389/fimmu.2025.1708467 (PMC12816181; doi:10.3389/fimmu.2025.1708467)
Supplement: Supplementary file 2 [file DataSheet2.pdf]

Speth, et. al Supplementary Table 2

Gene Symbol	Gene Name
Hexa	Beta-hexosaminidase A
Hexb	Beta-hexosaminidase B
Hif1a	Hypoxia inducible factor 1 alpha
Ldha	Lactate dehydrogenase A
Ldhb	Lactate dehydrogenase B
Acly	ATP citrate lyase
Hk2	Hexokinase 2
Idh1	Isocitrate dehydrogenase 1 (NADP+), soluble
Adpgk	ADP dependent glucokinase
Eno1	Enolase 1, alpha non-neuron
Erbb2	Erb-b2 receptor tyrosine kinase 2
Foxo3	Forkhead box O3
Gapdh	Glyceraldehyde-3-phosphate dehydrogenase
Gpi	Glucose phosphate isomerase 1
Gsk3b	Glycogen synthase kinase 3 beta
Hsf1	Heat shock transcription factor 1
Mtor	Mechanistic target of rapamycin kinase
Pdk1	Pyruvate dehydrogenase kinase, isoenzyme 1
Pdk2	Pyruvate dehydrogenase kinase, isoenzyme 2
Pfkfb2	6-phosphofructo-2-kinase/fructose-2,6-biphosphatase 2
Pfkfb3	6-phosphofructo-2-kinase/fructose-2,6-biphosphatase 3
Pfkfb4	6-phosphofructo-2-kinase/fructose-2,6-biphosphatase 4
Pfkp	Phosphofructokinase, platelet
Pkm	Pyruvate kinase M
Ppargc1a	Peroxisome proliferator-activated receptor gamma coactivator 1-alpha
Senp1	SUMO specific peptidase 1
Sod1	Superoxide dismutase 1
Stat1	Signal transducer and activator of transcription 1
Tgfb1	Transforming growth factor beta
Tnfsf13b	TNF superfamily member 13b
Vegfa	Vascular endothelial growth factor A
Vhl	Von hippel-lindau
